# Mass Spectrometry-Based Identification of MHC-Associated Peptides

**DOI:** 10.3390/cancers12030535

**Published:** 2020-02-26

**Authors:** Sachin Kote, Artur Pirog, Georges Bedran, Javier Alfaro, Irena Dapic

**Affiliations:** International Centre for Cancer Vaccine Science, University of Gdansk, 80-308 Gdansk, Poland; sachin.kote@ug.edu.pl (S.K.); artur.pirog@ug.edu.pl (A.P.); georges.bedran@phdstud.ug.edu.pl (G.B.); javier.alfaro@ug.edu.pl (J.A.)

**Keywords:** Neoantigens, mild acid elution, immunoprecipitation, mass spectrometry, cancer

## Abstract

Neoantigen-based immunotherapies promise to improve patient outcomes over the current standard of care. However, detecting these cancer-specific antigens is one of the significant challenges in the field of mass spectrometry. Even though the first sequencing of the immunopeptides was done decades ago, today there is still a diversity of the protocols used for neoantigen isolation from the cell surface. This heterogeneity makes it difficult to compare results between the laboratories and the studies. Isolation of the neoantigens from the cell surface is usually done by mild acid elution (MAE) or immunoprecipitation (IP) protocol. However, limited amounts of the neoantigens present on the cell surface impose a challenge and require instrumentation with enough sensitivity and accuracy for their detection. Detecting these neopeptides from small amounts of available patient tissue limits the scope of most of the studies to cell cultures. Here, we summarize protocols for the extraction and identification of the major histocompatibility complex (MHC) class I and II peptides. We aimed to evaluate existing methods in terms of the appropriateness of the isolation procedure, as well as instrumental parameters used for neoantigen detection. We also focus on the amount of the material used in the protocols as the critical factor to consider when analyzing neoantigens. Beyond experimental aspects, there are numerous readily available proteomics suits/tools applicable for neoantigen discovery; however, experimental validation is still necessary for neoantigen characterization.

## 1. Introduction: Neoantigens in Cancer Immunotherapy

Personalized immunotherapies and patient-specific methods emerged as an attractive alternative to the conventional methods and targeted therapies available to fight cancer [1,2]. Immune checkpoint inhibitors are used to modulate systems of T-cell regulation and some of them (e.g., ipilimumab) are now the standard of care for melanoma and a growing set of cancers exhibiting a high mutational burden. Furthermore, targeting of the cytotoxic T-lymphocyte-associated antigen (CTL4) immune checkpoint with ipilimumab (a human monoclonal antibody) improved overall survival of melanoma patients [3], and a combination of treatments that target the PD-1(Programmed cell death protein 1) checkpoint inhibitor resulted in more prolonged progression-free survival compared to ipilimumab alone [4].

However, some other immunotherapies consider more personalized treatments that target tumor-associated antigens (TAAs), which can serve as biomarkers and are overexpressed by the tumor cells, or with peptide neoantigens, which are derived from the tumor-specific DNA. Peptide epitopes present on the major histocompatibility complex (MHC) of the cancer cells [5,6] can uncover tumor-specific mutations and motifs. MHC molecules are encoded by human leukocyte antigen genes (HLA) which are the most polymorphic gene cluster in the human genome. Consequently, immunopeptidomes are a highly versatile group of peptides presented on the surface of the cells to T-cell receptors (TCRs), which, after their recognition, activate T-cells. However, a patient’s response to therapy is a complex event related to tumor mutations, as well as genetic and transcriptomic changes. Today, the emerging field of cancer proteogenomics integrates the information obtained from the genomic and proteomic studies toward identifying and validating peptide sequences as potential therapeutic candidates [7]. 

Currently there are continuous efforts to apply neoantigen-based therapies in real clinical settings, and ongoing clinical trials are mostly in phase I (https://clinicaltrials.gov/). Published cases focus on assessing the safety of the vaccines, as well as on the evaluation of the generation of CD4+ and CD8+ cells reactive to the neoantigens. This approach showed the efficiency of such vaccines in melanoma, which is characterized with high mutational burden [8,9]. Ott et al. [9] applied an in silico antigen prediction method with expression validation at the RNA level to compose a peptide library which was further used to formulate a peptide-based vaccine. Authors were able to confirm the immunogenicity, as well as show the promising patient outcomes, as four out of six patients did not experience recurrence after 25 months, and, in the remaining two cases, there was complete tumor regression after therapy with anti-PD-1. In cases of trials involving glioblastoma, which shows a substantially lower level of mutations, there was a detectable immune response, as shown by the presence of neoantigen-specific lymphocytes [10] and tumor-infiltrating lymphocytes [11]. Remarkably, Migliorini et al. [10] used antigen sequences which were directly derived from mass spectrometry-based immunopeptidome sequencing. In all published cases, the vaccines were well tolerated by patients. There is a high potential of integration of knowledge about neoantigens into other methods of cancer immunotherapy, such as endogenous T-cell therapy [12]. In this application, detected antigens are used to sort reactive T-cells, which can further be expanded in vitro and used for immunotherapy. However, despite the clear advantages of predicting neoantigens from genomic data, aberrations appear across each level of the central dogma. For example, at the transcript level, RNA-editing and aberrant splicing can introduce new sources of neopeptides. Furthermore, translational errors and post-translational modifications (PTMs) of those antigens that cannot be predicted can be characterized by mass spectrometry analysis. Moreover, PTMs were found to be more efficient in triggering an immune response. 

Mass spectrometry (MS) is an indispensable tool in proteome discovery and, as such, it is widely used in the clinic to profile patient samples and aid their treatment. Novel technological developments in MS improved targeting and identification of cancer-specific antigens as the basis of antigen-specific therapies. Sequencing of the immunopeptidome was pioneered by Hunt et al. in the 1990s [13]. Prediction of cancer-specific antigens is a challenge in immunotherapies [14], and the fact that the accurate identification of the peptide neoantigens can serve as the basis for personalized anti-tumor treatments makes mass spectrometry an essential tool in mapping the tumor immunopeptidome [14,15,16]. HLA-binding peptides were targeted in various cancer types of cell lines and tissues including melanoma, breast, and brain tumors [16,17,18,19,20]. Detection of the immunopeptidome may support the inclusion of tumor-associated neoantigens for the vaccine preparation, as well as expand the knowledge about the pathways leading to the peptide presentation on the cell surface.

Modern immunopeptidomics is a cutting-edge application [21]; the technical development in terms of the sample preparation, detection, and further computational identification of the peptides is still in development. Above all, low amounts of the neoantigens available per sample require improvements in the processing protocols and more efficacy of the current liquid chromatography–mass spectrometry (LC–MS) methods. Therefore, in this review, we discuss protocols for neoantigen isolation, as well as currently used LC–MS approaches for their detection.

## 2. MHC I and II Complexes

### 2.1. Isolation of the Immunopeptides from MHC Complexes

**Figure 1:**
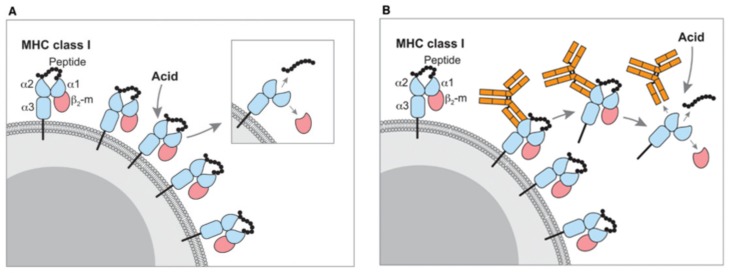
Methods for major histocompatibility complex (MHC) peptide isolation. (**A**) Acid wash and (**B**) immunoprecipitation (IP)-based purification. Reprinted with permission from Schumacher et al. [25], copyright year 2017, John Wiley and Sons.

In the last 30 years, the isolation of MHC-bound peptides (MBPs) was typically performed with two established methods named immunopurification/immunoprecipitation (IP) and mild acid elution (MAE) (Figure 1). In typical conditions, the identification of several thousands of MBPs requires more than 100 million antigen-presenting cells (APCs), which is the issue often encountered with a limited amount of the sample available from patients. 

In 1987, Sugawara et al. described a simple, reproducible method to eliminate the antigenicity of MHC I molecules called mild acid elution. The method used two minutes of incubation of the viable cells in a citric acid buffer (pH 3), and it was applied not only for humans but also for murine cells from various origins [22]. A crucial step in MAE is maintaining a pH of 3 for an effective elution of MHC I molecules. Moreover, the authors described that removal of HLA-A, B, and C antigenicity was less significant at pH higher than 3.7 and alkaline solutions at pH 8–11. An interesting observation from the MAE method is that neither MHC class II antigens nor other non-MHC antigens were removed from the cell surface [22].

Moreover, β2-microglobulin (β2M) is the component of the MHC I molecule present on the surface of the cell, and the MAE method facilitates dissociation of the extracellular non-covalently bound β2M from the MHC I complexes on the surface of the cell [23]. The MAE method was used to investigate the MHC I-presented peptide pool in different biological samples and was successfully applied for isolation and analysis of naturally presented MHC-associated viral peptides [24]. After isolation, the detection of MHC peptides was performed with reversed-phase high-performance liquid chromatography (RP-HPLC) coupled to tandem mass spectrometry. These techniques provided a successive experimental path to sequence the MHC peptides, with a significant impact on the cancer vaccine pipeline.

In another study, Storkus et al. used a single-cell suspension, incubated with a citrate-phosphate buffer for a short time (15 s) at a low pH (pH 3.3). Incubation in the citric buffer enabled swiping off the cell surface MHC-I-associated peptides while maintaining the cell viability, which was used for the identification of T-cell epitopes [18]. However, due to the stability of MHC-II–peptide complexes, this acidic treatment was unable to release MHC-II peptides [23].

The second, most successful, and well-known method for the isolation of MHC I and MHC II peptides is called immunopurification/immunoprecipitation (IP). IP is an antibody-dependent method based on the generation of robust and specific HLA-specific antibodies that recognize the pan-MHC or specific HLA alleles. This approach usually gives a higher number of MHC peptide identifications from the cell lines and clinical samples or tumor tissues [18,26,27,28].

Hunt et al. in their pioneering study investigated IP-based identification of peptides bound to MHC I molecules and their sequencing by MS [13] using a combined set-up of microcapillary high-performance liquid chromatography–electrospray ionization tandem mass spectrometry (HPLC–ESI-MS/MS). This set-up leads to sequencing a sub-picomolar amount of peptides from the MHC molecule, which enhances the identification of MHC-associated peptides for transformed and virally infected cells. Currently, advanced techniques such as nano-ultra-performance liquid chromatography coupled to high-resolution mass spectrometry (nUPLC–MS/MS) [28] and 96-sample plate high-throughput platforms [27] are essential improvements in the isolation of MHC-associated peptides and their detection. These methods consisted of multiple replicates of tissues (*n* = 7), with multiple cell lines (*n* = 21, 108 cells per replicate), and they showed high reproducibility (e.g., Pearson correlations for HLA I and II were up to 0.98 and 0.97, respectively) [27]. In parallel, significantly improved bioinformatic pipelines aided in the identification of a few thousands of MHC peptides in a short period [27,28].

The first step in the IP method is the solubilization of the tissues or cells and the cell surface of MHC complexes in a lysis buffer with a detergent. The solubilized MHC complexes are further captured by the pan-MHC I antibody on the principle of immunoaffinity (i.e., W6/32 antibody) that recognizes all alleles (i.e., HLA class I alleles A, B, and C). Furthermore, the formed antibody–MHC complexes are thoroughly washed to remove the unspecific bindings, detergents, and contaminants. After the washing procedure, MHC-associated peptides are separated from the antibody, MHC molecules, and β2M by solid-phase extraction using C-18 discs. Finally, purified peptides are subjected to the high-resolution MS. In the above-mentioned study, Lanoix et al. observed that IP typically provides 6.4-fold higher number of MHC I-associated peptides compared to the MAE method [26]. The authors also observed that most peptides identified by the MAE were also detected in extracts from the IP method.

In 2006, Gebreselassie et al. performed immunopeptidome analyses to compare MAE and IP methods on U937 cells and further used matrix-assisted laser desorption/ionization tandem time of flight (MALDI-TOF/TOF) MS for the identification of the MHC I-associated peptidome. The authors identified 64 and 21 MHC I-associated peptides by MAE and IP, respectively [29]. Later in 2014, Hassan et al. introduced isotopically labeled peptide MHC monomer (hpMHC), which they added directly to the cell lysate for accurate quantification of MHC class I-presented peptides. A combination of the hpMHC with IP-based peptide isolation method on B-LCL cells revealed the average recovery yield of 1%–2% on two minor histocompatibility antigens (MiHAs) [30]. As IP is one of the widely accepted methods to study the MHC expression mechanism, Komov et al. reported that expression of MHC I peptides depends on the availability of empty MHC molecules and not on the peptide supply [31]. Furthermore, for the comprehensive quantification of immunopeptidomes, Caron et al. introduced HLA allele-specific peptide spectral and assay libraries to extract the digital SWATH-MS data (sequential window acquisition of all theoretical mass spectra MS) for quantitative analysis of immunopeptidomes across several samples [32]. Very recently, in 2018, Lanoix et al. applied both MAE and IP methods for the comparative identification of the MHC I immunopeptidome repertoire of B-cell lymphoblasts [26]. Particularly in the MAE approach, the viable cells (B-LCL) were incubated with citrate buffer (pH 3.3) to disrupt the MHC I complex; hence, β2M protein and MHC-associated peptides were released into the MAE buffer. Furthermore, the peptides were desalted, and β2M was removed via the ultrafiltration method. Subsequently, the eluted purified peptides were subjected to MS analysis. Here, with the MAE approach, the authors identified few hundreds to few thousands of MHC-associated peptides (Table 1) [26]. 

The exact source of targeted tissue-specific antigens and MHC expression pathways is still unclear. Moreover, there are many potential sources of MHC-associated antigens such as classical MHC pathways, pioneer translation products (PTPs) [41], defective ribosomal products (DRiPs) [42], cis- and trans-spliced peptide [43], non-canonical reading frames [44], etc. Therefore, the combination of high-resolution MS and MHC peptide isolation methods (MAE and IP) is not only convenient for accurate identification of MHC peptide neoantigens; it also indirectly contributes to detecting the source of origin at a genomic location and to tracing the exact mechanism of MHC peptide expression, which is a pressing issue [40]. The use of the MAE method due to the high number of contaminating peptides limits the possibility of doing an in-depth analysis of the immunopeptidome. On the contrary, IP facilitates the direct and in-depth analysis of clinically relevant neoepitopes on human tissue by MS [18]. Therefore, while the IP method shows some more convenience for use over the MAE method, both methods have their particular advantages in terms of the duration of the protocols, specificity for immunopeptides, and achievable throughput, which all aid in identifying relevant neoantigens to answer biological questions [16,45]. Therefore, it is recommended that both methods can be applied in parallel to gain a maximal amount of biological information and to understand the dynamic nature of the immunopeptidome. In Table 2, we summarize a comparison of MAE and IP methods with highlighted advantages and drawbacks of each technique.

### 2.2. Detection and Identification of Immunopeptides with Mass Spectrometry

Until recently, most efforts in neoantigen identification were performed with indirect methods, relying on genome and transcriptome sequences [18,46,47]. This information can be used to detect mutations and confirm the transcription of the variant DNA sequences. Then, it can be further analyzed to predict the probable neoantigen sequence [5], affinity to MHC molecules [48], and immunogenicity [49]. However, due to the vast amount of possible neoepitopes, additional confirmation at the protein level may be used to further narrow down the list of potential neoantigens [37].

Currently, LC–MS/MS-based immunopeptidomics is the only method that can comprehensively interrogate the repertoire of MBPs presented in vivo. The idea behind the use of MS for neoantigen discovery relies on direct detection of the sequences of neoantigens presented on the cell surface [50]. Currently, great efforts are made to make this possible, as well as to make the workflows reliable enough for application of MS-based neoantigen identification in the clinical setting [51,52].

In most of the methods used today, the starting amount of the material required for in-depth LC–MS analysis is about 10^8^ cells [28] or 1 g of the tissue sample [17]. In such cases, the amount of isolated immunopeptides should enable identification of several thousands of individual MHC ligands. After the isolation from the MHC complexes, immunopeptides are further separated by nanoscale LC. These separations of the MHC-bound peptides are carried out in a way partially similar to the typical proteomic experiments, with several differences. Firstly, the gradient used for immunopeptides is usually shallower, utilizing less than 30% acetonitrile. Secondly, the analysis time used for immunopeptides is usually longer compared to the time used for tryptic peptide separation, which leads to the use of long columns with high peak capacity. Usually, immunopeptides are analyzed without prefractionation, due to possible sample losses; however, if enough of starting material is available (probably limiting the scope to the cell culture samples), prefractionation through strong cation exchange (SCX), high-pH reverse-phase chromatography, or isoelectric focusing (IEF) can lead to a substantial increase in the number of identified peptides [53,54]. As these studies reported a vast improvement of the identification number after prefractionation, it is expected that single-step separation methods are currently not sufficient to cover the complexity of the immunopeptidome.

**Figure 2:**
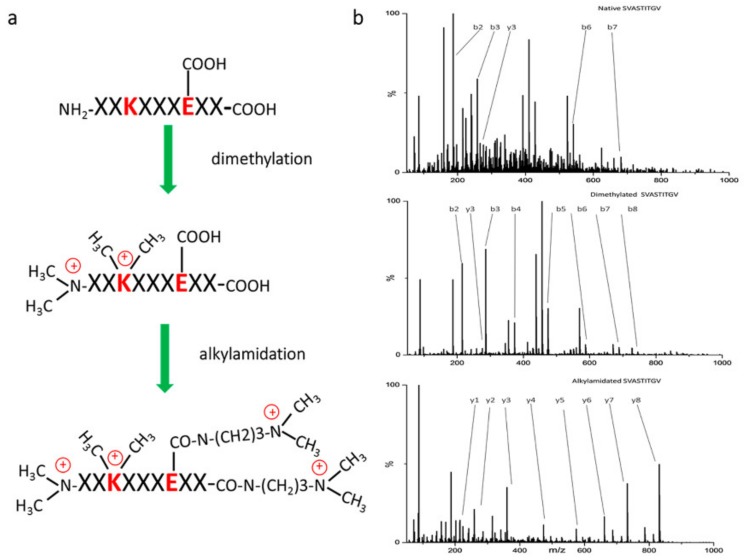
Derivatization reaction to increase sequence coverage and ionization efficiency of peptides. (A) Scheme of the derivatization and (**B**) tandem MS spectra of synthetic peptide (SVATITGV) in native, dimethylated, and alkylamidated form. Reprinted with permission from Chen et al. [39], copyright year 2018, American Chemical Society.

Reports showed that the MBPs have different physiochemical characteristics than typical tryptic peptides. The difference occurs from the fact that they do not always contain a basic residue on the C-terminus [55]. Because of the absence or reduction in the basic residues, effective ESI ionization and fragmentation may be hampered. One possibility to overcome this issue is chemical derivatization of the functional groups of the immunopeptides. There were attempts to increase their identification rate via chemical modifications since the inception of immunopeptidomics. As an example, the *N*-pirydylacetyl modifications of amino groups was shown to increase the completeness of b-ion series [56]. More recently, Chen et al. described two different chemical derivatization approaches for extending the identification of MBPs. Firstly, peptides were dimethylated on all amino groups including N-termini, which does not significantly affect their fragmentation but increases hydrophobicity and, therefore, improves their chromatographic properties. Secondly, they were alkylamidated on the carboxyl groups including C-termini. This modification affects the fragmentation pattern, leading to the generation of more complete y-ion series, increasing the sequence coverage (Figure 2) [39]. Analysis of the native, dimethylated, and alkylamidated peptides from the same sample enabled the detection of a subset of previously undetected peptides and, thus, led to a substantial increase in immunopeptidome coverage.

In immunopeptidomics, mass spectra are almost exclusively collected on fast, high-resolution instruments employing Orbitrap or time-of-flight analyzers for discovery-oriented experiments. Even after decades of research, the overall complexity of the immunopeptide identification represents a great challenge in terms of performance and reproducibility of the analyses. The high complexity of the sample, along with lack of clear sequence specificity of MHC ligands makes obtaining a high-quality MS/MS spectrum crucial for peptide identification. The most utilized fragmentation strategy is the use of higher-energy collision-induced dissociation (HCD) or collision-induced dissociation (CID), which are both capable of generating MS/MS spectra at high speed. Moreover, the peptide antigen sequence coverage is further improved by alternative fragmentation strategies such as electron capture dissociation (ECD) or electron transfer dissociation (ETD) and HCD combinations [57], which are particularly useful with de novo sequencing approaches that exclusively rely on fragmentation data to assign peptide sequences. Electron-transfer/higher-energy collision dissociation (EThcD) fragmentation was applied on an Orbitrap Velos instrument with modified firmware, and it was shown to generate fragmentation patterns with high sequence coverage. This method relies on all-ion HCD fragmentation of ETD fragmentation products and generates *b, c, z,* and *y*-type ions [58]; however, the tradeoff is the lower acquisition speed compared to conventional HCD [57].

Examples of immunopeptidomics studies with details on the most important parameters, such as the type of the column and instrument, amount of the starting material, and obtained main results, are summarized in Table 2. Moreover, confirmation of the presence of a particular peptide or antigen quantitation can also be done on triple quadrupole or quadrupole-ion trap type instruments [34,59]. The application of such analyses is especially useful for highly reproducible peptide detection and quantitation.

One of the novel challenges of the immunopeptidomics is the characterization of the post-translationally modified MHC ligands. Analysis of glycosylated [60] and phosphorylated [61,62] MHC ligands shows that these modifications are realistic and have an influence on the immunogenicity. Detection and characterization of glycosylated MHC II ligands is feasible using combined HCD and ETD fragmentation techniques, even without affinity enrichment [60]. The phosphorylated peptides constitute approximately 1–2% of total antigens, and there are sequence motifs specific for phosphorylated ligands [63]; however there is a wide variety of other possible modifications such as acetylation, methylation, citrullination, and cysteinylation [64]. Detection of the PTMs further increases the complexity of the pipeline, and analysis of neopeptide PTMs is hampered because of the increase in the already big search space by additional possibilities [65].

The analysis of the obtained immunopeptidome LC-MS data is challenging compared to most proteomics experiments. The most popular data analysis strategy in peptide-centric proteomics is a database search that matches a theoretical fragment spectrum from a candidate peptide sequence to an observed fragment spectrum. Typically, candidate peptides originate from an in silico digested protein database and are selected for comparison based on the mass of the precursor ion. This differs in neoantigen discovery, where the whole analysis workflow is highly dependent on the previously acquired genomic data, as the sequence database must contain all the mutations expected in the neoantigen sequences. Mutations and aberrations can be called from sequencing data using standard measures, currently well optimized by the genomics community [66,67,68,69]. To date, most immunopeptidomics studies still use the conventional whole proteome database search strategy with search engines such as MaxQuant, PEAKS, SEQUEST and Proteome Discoverer [27,70,71], in combination with oxidation (M), deamidation (NQ), N-terminal acetylation, and phosphorylation (STY) as variable modifications. Due to the fact that the cleavage specificity is still unknown, in silico protein digestion is performed in a nonspecific manner, penalizing both the search performance and the quality of the search results. 

After peptide-spectrum matching, post-processing pipelines also differ from those used in global proteomics. The lack of protein level information reduces overall confidence in correct identifications. Each peptide must be scored individually, irrespective of its source protein [21]. For these reasons, an immuno-peptidomic post-processing algorithm called MS-rescue [72] for refining the spectrum-peptide assignments was developed. It was designed specifically for MHC studies, achieving an increased sequencing depth, while at the same time removing potential experimental outliers and contaminants. For the same reasons, some argue that using a biologically relevant peptide database rather than the whole proteome would significantly speed up the searches and increase sensitivity. For instance, SpectMHC [73] implements a targeted database search approach showing a two-fold increase in peptide identifications compared to unspecific proteome search.

Mass spectrometry lags behind in terms of the development of file standards, when compared to genomics or transcriptomics. Other than the MIAPE initiative (minimal information about immunopeptidomics experiment), there is a need for standardized and version-controlled bioinformatics workflows, especially when considering potential clinical applications. One of the examples of such a workflow is MHCQuant [74], which also shows superior identification capabilities compared to other identification engines, as well as support for label-free quantitation. Remarkably, authors showed the identification and confirmation of previously undetected neoantigens in a publicly available dataset. Probabilistic inference of codon activities by an EM algorithm (PRICE), another MHC-related workflow [75], is a computational method for accurately resolving overlapping small open reading frames (sORFs) and noncanonical translation initiation sites, revealing it as a substantial fraction of the antigen repertoire. 

Complete de novo peptide sequencing is required to identify a part of the immunopeptidome. The recently discovered peptide splicing phenomenon could lead to the presentation of neoantigens of sequences that are not present in the genome. The portion of spliced peptides in the immunopeptidome is not known, but some reports suggest that it may be up to 30% [76]. The *de novo* sequencing of HLA ligands cannot be done manually, due to the large amount of created data; therefore, recently, automated tools started to become available with examples of the most popular software tools as PEAKS and PepNovo+ [77]. There was significant progress in this field in recent years due to the availability of MS instruments capable of high-speed acquisition with high-resolution MS/MS spectra, as well as developments in computational methods, such as the introduction of deep learning-based workflows for peptide sequencing. For example, DeepNovo uses the peptide sequences identified via database searching to train a personalized de novo sequencing algorithm [78]. Similarly, SMSNet [79] is a deep learning-based de novo sequencing model with a post-processing strategy that pinpoints misidentified residues and utilizes a sequence database to revise the identifications. In general terms, the hybrid de novo/database approach relying on mass tags (partial peptide sequence inferred from a spectrum, which is used to search the database with total peptide mass as a constraint) or the use of prior knowledge of the predicted MHC-binding motifs to further filter out the improbable peptide sequences and perform rescoring to rescue probable hits of lower quality may improve the sensitivity and accuracy of the searches [18,72].

Beyond the direct identification of immunopeptides by mass spectrometry, a variety of predictive software exists to identify those genomic variants that would be presented to the immune system. There are several tools to process genomics and transcriptomics data to identify potential neoantigens. These include, for example, PVacTools [80,81,82] and Neopred Pipe [83] These tools can be used to establish mutations likely present in the immunopeptidome, as well as to narrow down those that have affinity for MHC. The best way to integrate the results of these tools, which are of course limited in their ability to predict correctly, with personalized and directly measured immunopeptidomes remains an open question.

## 3. Conclusions

The characterization of peptides presented as antigens on the cell surface is of vital interest to our understanding of immune-related diseases including pathogen response, auto-immune diseases, and cancers. Cancers are of particular interest as they rely on creating an immune-suppressive environment as one of their hallmarks. In the end, the direct detection of peptides will be hugely important for our understanding of biology and to identify candidates for immunotherapeutic intervention in this disease. The direct detection of these peptides is essential, as current neoantigen prediction methodologies from genomics and transcriptomics are error-prone. Nevertheless, direct detection by mass spectrometry suffers from sensitivity issues and complicated sample preparation strategies, which burdens its broad adoption. Isolation and purification of the immunopeptides is one of the most important steps in their analysis. Further development of the sample preparation protocols to improve isolation of MHC peptides and reduce the number of preparation steps might minimize the loss of low abundant peptides. Moreover, limited patient sample amounts affect the sensitivity and reproducibility of detection, which are major challenges in the MS discovery of immunopeptides. Chemical derivatization approaches might aid in bettering chromatographic performance and ionization efficiency of the peptides, thus extending their sequence coverage and identification. The next decade will see tremendous advantages in neoantigen characterization by mass spectrometry fueled by increased instrument sensitivity, thus reducing tissue quantity requirements.

## Figures and Tables

**Table 1:** Summary of selected immunopeptidomic studies and their comparison in terms of isolation method and instrumental detection. EBV—Epstein-Barr virus DMSO—dimethyl sulfoxide.

Study Type	Cancer Type	Isolation Method	LC	Mass Spectrometer	Data Analysis	Sample Type and Amount	Number of Identified Peptide Ligands	Identified Neoantigens	Validation Method	Study
Neoantigen discovery	B-cell lymphoma	IP of MHC-I and MHC-II	180 mm × 100 µm C18 column, 160 min gradient, DMSO as mobile phase additive	LTQ Orbitrap Elite, Combined CID+HCD	SEQUEST and PEAKS DB for database searching, PEAKS Studio de novo sequencing	10^8^–10^9^ cells	>24,000 MHC-I >12,000 MHC-II	14	MHC affinity, existence of specific T-cells in patients	[33]
Confirmation of in-silico discovered neoantigens	MCA sarcoma cell lines	IP of MHC-I	Discovery: 500 mm × 50 µm C18 column, 140 min gradient, targeted: 150 mm × 75 µm C18 column, 60 min gradient	Discovery: LTQ Orbitrap XL Targeted: AB Sciex QTRAP 5500	Discovery: MASCOT Targeted: SEQUEST, Skyline	5 × 10^8^ cells	250 MHC-I	1 confirmed	MHC tetramer staining, immunogenicity	[34]
Neoantigen discovery	MC-38 and TRAMP-C1 mouse tumor cell lines	IP of MHC-I	250 mm × 75 µm column packed with 1.7 µm C18, 180 min gradient	LTQ-Orbitrap Velos	Mascot	1 × 10^8^ cells	6239 for MC-38 3631 for TRAMP-C1	7 MC-38 0 TRAMP-C1	In vivo immunogenicity	[35]
Neoantigen discovery	Meth A mouse cell line	IP of MHC-I	450 mm × 75 µm column packed with 1.9 µm C18, 85 min gradient	Q-Exactive HF, Q-Exactive HFX	Discovery: MaxQuant. PRM verification: Skyline	1–10 × 10^8^ cells	6209	8 confirmed	Spike-in heavy peptides, in vivo tumor rejection	[36]
Neoantigen discovery	Melanoma tissue	IP of MHC-I	450 mm × 75 µm column packed with 1.9 µm C18, 90 min gradient	Q-Exactive, Q-Exactive HF	MaxQuant	25 tissue samples ranging from 0.1 to 4 g	78,605 MHC-I 15,009 MHC-II	11	In vitro immunogenicity	[18]
Neoantigen discovery	Hepatocellular carcinoma tissue	IP of MHC-I and MHC-II	250 mm × 50 µm PepMap RSLC (2um C18) column, 90 min gradient	LTQ Orbitrap XL	MaxQuant	Not exactly specified	average 1403 ± 621 from single sample	0		[37]
Comparison of purification methods	B lymphoblasts EBV transformed and leukemia xenografts	IP of MHC-I and MAE	100 mm × 150 µm column packed with 3 µm C18, 56 min gradient	Q-Exactive HF	PEAKS	2 × 10^6^, 2 × 10^7^, 10^8^ cells	2 × 10^6^: 314 MAE 2016 IP 2 × 10^7^: 2081 MAE 3931 IP 10^8^: 2996 MAE 5093 IP	not a subject of study		[26]
Neoantigen discovery	Colorectal cancer organoids	IP of MHC-I and MHC-II	500 mm × 75 µm column with 1.9 µm C18 material. 125 min gradient for MHC-I and 90 min for MHC-II peptides	Q-Exactive HFX	MaxQuant	3.85 × 10^7^–10^8^ per biological replicate. 42 biological replicates in total	average 9936 from single sample	3	None	[38]
Neoantigen discovery	HCT 116 cell line	MAE	500 mm × 75 µm column packed with 1.9 µm C18 material, 120 min gradient, chemical peptide derivatization	Q-Exactive	Mascot	2–6 × 10^8^ cells per replicate, total 9 technical replicates	3148 for 3 technical replicates	10	None	[39]
Neoantigen discovery	EL4, CT26 cell lines, primary leukemia and lung cancer cell lines	MAE	150 mm × 150 µm column packed with C18 material, 56min gradient	Q-Exactive Plus, Q-Exactive HF	PEAKS	2.5–7 × 10^8^ cells per sample. 17 samples in total	1875 CT26 cells 873 r EL4 cells	4 (CT26) 2 (EL4) 2 (human samples)	Synthetic peptides, selected hits for in vivo immunogenicity	[40]

**Table 2:** Comparison of advantages and drawbacks between mild acid elution (MAE) and immunoprecipitation (IP) methods. HLA—human leukocyte antigen.

Neoantigen Isolation Method		Reference
**Advantages**	Drawbacks	
Immunoprecipitation (IP)		
Due to specificity of the antibody, IP is less likely to select contaminating peptides	Due to the binding specificity of the antibody, HLA subtype information is also lost in IP-based methods utilizing the pan MHC antibody	[25]
Highly specific because the MHC antibodies capture the MHC complexes, where about 90% of MHC peptides come from immunopeptidomes	IP requires extensive washes to remove contaminants/unspecific peptides and detergent, which may lead to losses of low-affinity MHC I-associated peptides	[16,29]
Applicable for a variety of biological samples; fresh or frozen dissociated cells and solid tissues		[26]
Mild acid elution (MAE)		
MAE is applicable when starting material may be limited	After MAE, further analysis is challenging due to the additional cell surface (non-MHC) contaminating peptides bound through hydrostatic interactions that may also be eluted by this acid-wash process	[23]
MAE allows for cell regeneration following β2M-dissociation, so it can be re-analyzed after a second perturbation		[23]
This method is simple, quick, and reproducible	It extracts not only MHC I-associated peptides but also other contaminants or contaminant peptides	[22,38]
The MAE method can be used in situations where antibodies against MHC I molecules are not available	MAE needs viable dissociated cells to perform acid elution, which is the limitation when using fragile cells/solid tumor tissue	[45]
It involves fewer purification steps and no detergents	It is been used for high-throughput sequencing of the MHC I-associated peptide repertoire because it is assumed that eluted peptides contain not only MHC I-associated peptides, but also “contaminant” peptides	[45]
MAE introduces no bias linked to preferential loss of low-affinity peptides		[45]
